# Recent Advances in Molecular Imaging

**DOI:** 10.1155/2007/73198

**Published:** 2007-12-31

**Authors:** Jie Tian, Wenxiang Cong, Jing Bai, Ming Jiang, Wei Liang

**Affiliations:** ^1^Institute of Automation, Chinese Academy of Sciences, Beijing 100080, China; ^2^Life Science Center, Xidian University, Xian, Shaanxi 710071, China; ^3^Biomedical Imaging Division, VT-WFU School of Biomedical Engineering and Sciences, Virginia Polytechnic Institute & State University, Blacksburg, VA 24061, USA; ^4^Department of Biomedical Engineering, Tsinghua University, Beijing 100084, China; ^5^LMAM, School of Mathematical Sciences, Peking University, Beijing 100871, China; ^6^Institute of Biophysics, Chinese Academy of Sciences, Beijing 100101, China

Molecular imaging is a newly emerging and rapidly developing biomedical imaging field in which the modern tools are being married to depict noninvasive in vivo cellular and molecular processes sensitively and specifically, such as monitoring multiple molecular events, cell trafficking and targeting. The goals of this field are to develop technologies and instruments for studying biological and medical processes as well as diagnosing and managing diseases better. Although rapid progress in the fundamentals and applications make molecular imaging become an important tool for biomedical research in recent years, many difficult problems and challenges remain. Discussing the problems and challenges in detail and illustrating recent progress and future direction, the special issue collects the high-quality, peer-reviewed, original research papers in the area of molecular imaging.

Novel molecular imaging theories and algorithms, new molecular probes, multimodality molecular imaging prototype systems and experiments, and final clinical applications are introduced mainly in this special issue. In molecular imaging theories and algorithms, genetic algorithm-based optimization tool is used to improve the accuracy of the diffusion model in strongly absorbing media by adjusting the optical parameters. Furthermore, a penalized linear and nonlinear combined conjugate gradient method, a fast preiteration algorithm based on the generalized inverse matrix, and a Monte-Carlo-based network method are also proposed for light source reconstruction in optical tomography. Considering the importance of molecular probes, synthesis and bioconjugation of gold nanoparticles as potential molecular probes for light-based imaging techniques are described in this special issue, and the nitroimidazole-based thioflavin-T derivatives as cerebral ischemia markers are evaluated in vivo. In order to test the feasibility and effectiveness of imaging theories, algorithms and probes, molecular imaging prototype systems should be designed, constructed and employed for small animal or phantom imaging. Thus, multimodality fusion near-infrared optical tomography Systems with highly sensitive CCD camera and photomultiplier tube can be consulted respectively in this special issue. Furthermore, a novel confocal optical system design and a dual laser confocal scanner have been developed for molecular imaging applications of biochip. In clinical applications, the advantage of PET and CT integration in examination of lung tumors is analyzed. Moreover, several innovative processing methods of molecular image are also presented in this special issue. In conclusion, this special issue covers recent important advances in molecular imaging field.

